# CPEB2 Is Necessary for Proper Porcine Meiotic Maturation and Embryonic Development

**DOI:** 10.3390/ijms19103138

**Published:** 2018-10-12

**Authors:** Barbora Prochazkova, Pavla Komrskova, Michal Kubelka

**Affiliations:** 1 Department of Veterinary Sciences, Czech University of Life Sciences, Kamycka 129, 165 00 Prague, Czech Republic; Barbora.Prochazkova@eli-beams.eu; 2 Institute of Animal Physiology and Genetics, Czech Academy of Sciences, Rumburska 89, 277 21 Libechov, Czech Republic; pavla.komrskova@gmail.com

**Keywords:** oocyte maturation, embryonic development, translational control, CPEBs, CPEB2

## Abstract

Oocyte meiotic maturation and embryogenesis are some of the most important physiological processes that occur in organisms, playing crucial roles in the preservation of life in all species. The post-transcriptional regulation of maternal messenger ribonucleic acids (mRNAs) and the post-translational regulation of proteins are critical in the control of oocyte maturation and early embryogenesis. Translational control affects the basic mechanism of protein synthesis, thus, knowledge of the key components included in this machinery is required in order to understand its regulation. Cytoplasmic polyadenylation element binding proteins (CPEBs) bind to the 3′-end of mRNAs to regulate their localization and translation and are necessary for proper development. In this study we examined the expression pattern of cytoplasmic polyadenylation element binding protein 2 (CPEB2) both on the mRNA (by real-time quantitative reverse transcription polymerase chain reaction, qRT-PCR) and protein (by Western blotting, WB) level, as well as its localization during the meiotic maturation of porcine oocytes and early embryonic development by immunocytochemistry (ICC). For the elucidation of its functions, CPEB2 knockdown by double-strand RNA (dsRNA) was used. We discovered that CPEB2 is expressed during all stages of porcine meiotic maturation and embryonic development. Moreover, we found that it is necessary to enable a high percentage of oocytes to reach the metaphase II (MII) stage, as well as for the production of good-quality parthenogenetic blastocysts.

## 1. Introduction

The meiotic maturation of female germ cells, also known as oocytes, is characterized by the virtual absence of transcription. Therefore, the accumulation of all the necessary RNAs during oocyte growth is crucial for proper meiotic maturation and early embryonic development. In other words, early development is directed by maternally inherited mRNAs, which are synthesized and stored during the long period of oogenesis.

The main regulatory mechanisms of gene expression in oocytes therefore include the post-transcriptional modifications of mRNAs, which affect their translation. Translational regulation is partly controlled by changes in mRNA poly(A) tail length. Cytoplasmic polyadenylation mediates the translational activation, whereas poly(A) tail shortening from a defined starting point acts as a timer of mRNA degradation or silencing. Length control of mRNAs 3′UTR is important for numerous reasons. Translational regulation through cytoplasmic polyadenylation and deadenylation is essential for the meiotic maturation of oocytes. The correct balance between them is also critical for proper division. In fact, the sequential waves of polyadenylation and deadenylation drive the meiotic progression forward, preventing the incorrect timing of specific protein translation during particular maturation phases in oocytes. Default deadenylation in maturing oocytes could be a means to liberate components of the translation apparatus for the synthesis of meiosis-specific proteins [1,2,3,4,5,6,7,8].

The timely, specific expression of mRNAs is generally regulated by the variety of RNA-binding proteins. The most important of them are the cytoplasmic polyadenylation element binding proteins (CPEBs), which, in concert with other RNA-binding proteins, modulate the poly(A) tail length of mRNAs [9]. The specific poly(A) tail elongation and shortening are mediated by interactions of CPEBs with *cis* elements in mRNAs, namely the cytoplasmic polyadenylation element (CPE) [10]. The classical (canonical) CPE is defined as UUUUUAU; however, Du and Richter [11] revealed possible variations in this sequence. 

Four *CPEB* genes have been described in vertebrates (*CPEB1–4*), whereas only two *CPEBs* were identified in *Drosophila* (*Orb1–2*). *CPEB1* and *Orb1* genes are classified in a separate *CPEB* subfamily. Other members—*CPEB2*, *CPEB3*, *CPEB4*, and *Orb2*—belong to the *CPEB2* subfamily. Moreover, they are paralog proteins that are homologous among themselves. The homology between the CPEB proteins is limited to their C-terminal region, which contains two RNA-recognition motif (RRM) domains and one zinc finger structure. In contrast, the N-terminus differs greatly [12,13,14,15].

CPE-binding protein 1 (CPEB1) is one of the most studied RNA-binding proteins. It is well known as a polyadenylation promoter. CPEB1 was first described in *Xenopus* oocytes, where it is involved in the regulation of maturation [10]. It has also been reported to be an important factor for the regulation of the synaptic function of the mouse brain [16].

During female meiosis, CPEB1 plays a significant role in the formation of the synaptonemal complex through the regulation of the mRNAs for *SCP1* and *SCP3*. CPEB1 phosphorylation initiates the translation of *cyclin B1* and *mos proto-oncogene* (*c-mos*) mRNAs, which are essential for the progression to MII stage oocyte and the establishment of cytostatic factor (CSF) mediated arrest [17]. CPEB1 was later reported to be involved in the regulation of several of the initial embryonic mitotic divisions as well as cell cycle progression [18,19,20,21,22].

Like CPEB1, the proteins from the CPEB2 subfamily participate in translational regulation. They bind CPEs and other recognition elements in the 3′-UTR of specific target mRNAs [12]. The specific binding of CPEB2 to a CPE has been verified by a lack of binding to an mRNA fragment without a CPE, as well as by the fact that CPEB2 binding to the CPE-containing fragment occurred with homopolymeric poly(U) RNA, but not with poly(A) or poly(C) [23,24]. As an example, in the case of *hypoxia inducible factor 1, subunit α (HIF-1α*) mRNA, the sequence in the 3’UTR, to which the CPEB2 probably binds, is UUUUCAU [25]. In the case of this mRNA, another type of regulation of translation through CPEB2 has been described. CPEB2 slows down the elongation phase of *HIF-1α* translation by connection with the eukaryotic elongation factor eEF2 [25].

The proteins from the CPEB2 subfamily are expressed abundantly and are generally present in the nervous system and in the germline. CPEB3 is expressed in the brain [13], while CPEB4 is abundant in oocytes and early embryos [26]. In mice, *CPEB2* mRNA is profusely expressed in male germ cells (its mRNA being first detected in post-meiotic early spermatids) and the brain, but it can be detected by RT-PCR in all tested mouse tissues, including ovarian tissue. CPEB2 is also detected in the cytoplasm of HeLa cells [27]. Moreover, Johnson et al. [28] demonstrated that the regulation of *CPEB2* mRNA splicing is a key mechanism in anoikis resistance and a driving force in triple negative breast cancer metastasis. However, the importance of CPEB2 in female reproduction processes has not yet been explored.

To extend the knowledge of the roles that CPEB-related proteins play in reproduction, we focused on the expression and function of CPEB2 during porcine meiotic maturation and early embryonic development.

## 2. Results

### 2.1. Expression of CPEB2 mRNA during Meiotic Maturation of Porcine Oocytes and Early Embryonic Development

**Figure 1:**
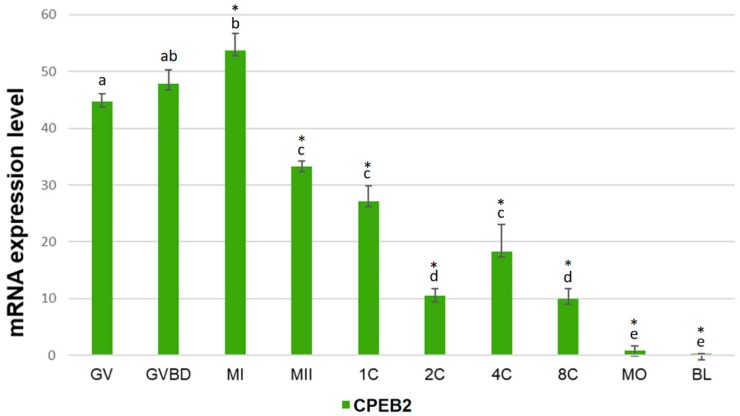
Expression of CPEB2 mRNA during oocyte maturation and early embryonic development. The relative abundance CPEB2 mRNA was determined by qRT-PCR in porcine oocytes and parthenogenetic early embryos including GV, GVBD, MI, and MII oocytes, pronuclear (1C), two-cell (2C), four-cell (4C), eight-cell (8C), morula (MO), and blastocyst (BL) embryos. CPEB2 transcript levels were normalized relative to the abundance of GAPDH mRNA (exogenous control) and are shown as means ± SEM (*n* = 4 independent biological experiments). Different letters indicate statistical difference (*p* < 0.05), * indicates statistical difference compared to the mRNA level in the GV stage (*p* < 0.05).

The expression of CPEB2 mRNA during the meiotic maturation of porcine oocytes and embryonic development was studied by qRT-PCR. We used a set of primers towards the central part of CPEB2 open reading frame (ORF). The relative positions of all primers used in this work are specified in Supplementary Figure S1. During the meiotic maturation, the amount of CPEB2 mRNA increased gradually between GV (germinal vesicle), GVBD (germinal vesicle breakdown), and MI (metaphase I) phases, reaching a maximum in the latter phase. After the MI phase, the expression of CPEB2 mRNA dropped sharply. After parthenogenetic activation, CPEB2 mRNA levels decreased to reach a local minimum at the two-cell stage. The levels of CPEB2 mRNAs rose from the two-cell to the four-cell stage before decreasing to barely detectable levels in the blastocyst stage (Figure 1).

**Figure 2:**
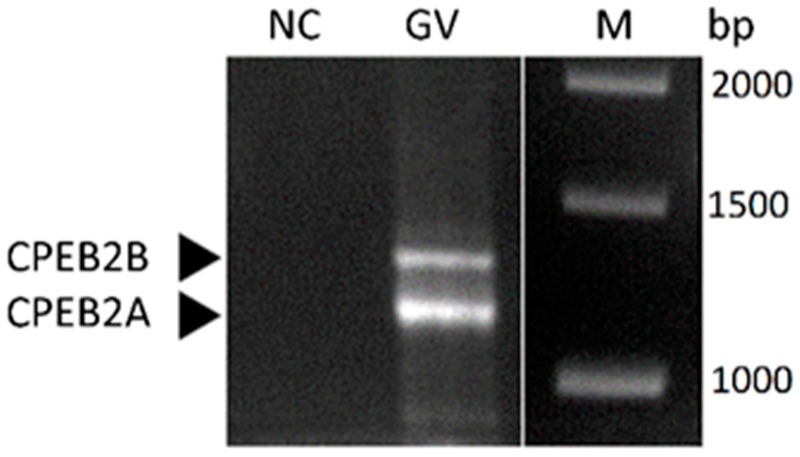
Two splice variants of CPEB2 transcript in porcine oocytes. CPEB2A (short variant) and CPEB2B (long variant) were detected using RT-PCR. GV = oocyte stage, NC = negative control, M = marker, bp = marker size. The experiment was repeated three times, with 280 oocytes per experiment.

Using a different set of primers, which enabled the amplification of the almost full-length ORF for RT-PCR (Figure S1), we detected two splice variants of CPEB2 transcript to be present in porcine oocytes (Figure 2). The length of the observed PCR products corresponds to the CPEB2 variants A and B, which were previously published in NCBI [29,30]. CPEB2A is a shorter variant that lacks 90 nucleotides in the coding region and encodes a 47.6 kDa protein in comparison to CPEB2B, which encodes a 50.8 kDa protein. However, the partial sequencing confirmed only the mRNA corresponding to the longer CPEB2B splice variant to be present in porcine oocytes (data not shown). 

### 2.2. Expression of CPEB2 Protein during Meiotic Maturation of Porcine Oocytes

**Figure 3:**
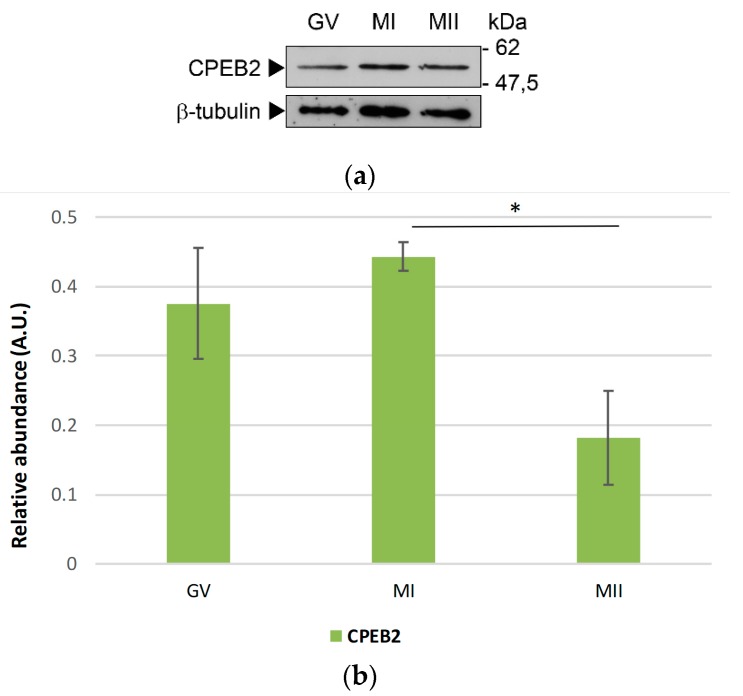
Relative protein expression of CPEB2 during the meiotic maturation of porcine oocytes. (**a**) Representative images from WB probed by antibodies for CPEB2 and β-tubulin proteins in the GV, MI, and MII oocytes. β-tubulin was used as a loading control. The experiment was performed three times, with 100 oocytes per experiment. (**b**) The protein expression of CPEB2 from three independent experiments was quantified using Quantity One software. The density of each individual band was normalized to the total density of the examined bands and to β–tubulin. The value of CPEB2 was summed. The values represent the means ± SEM and * indicates statistical difference (*p* < 0.05).

To investigate the CPEB2 protein expression in porcine oocytes, we used Western blotting with the antibody directed towards the N-terminal region of CPEB2. Regarding the two splice variants, the antibody used for this experiment does not discriminate between CPEB2A and 2B. We detected only one band of approx. 50 kDa in all stages of the meiotic maturation (Figure 3a and Figure S2) with a maximum abundance in the MI stage. The protein remained present until metaphase II, when its expression decreased significantly (Figure 3b).

**Figure 4:**
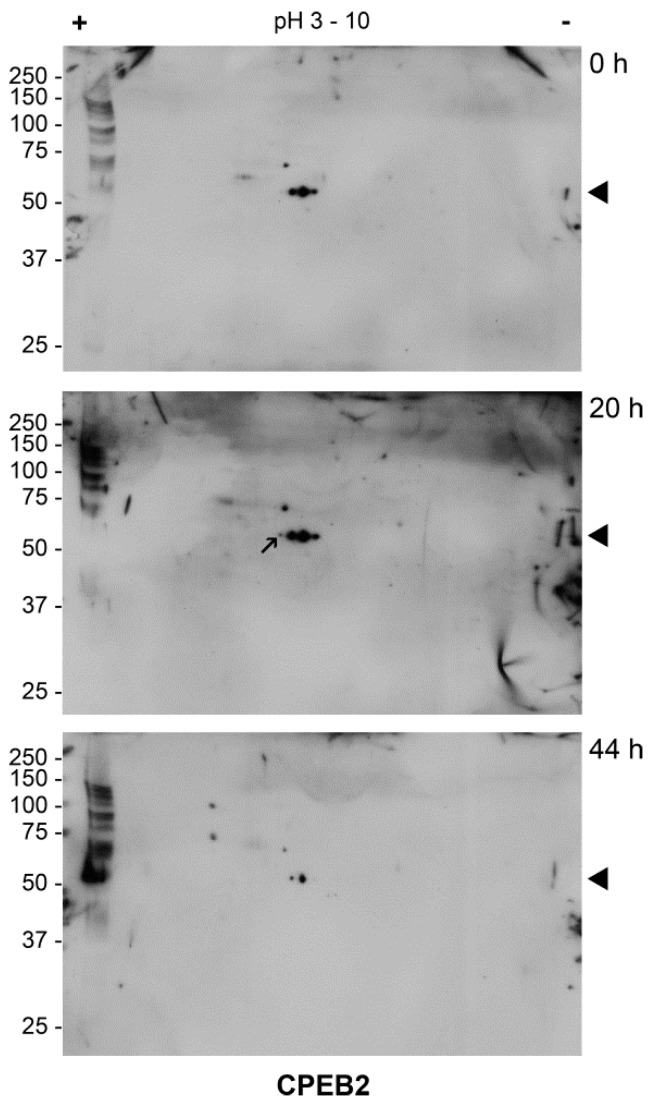
Detection of post-translational modifications of CPEB2 protein by 2DE Western blot. Two hundred immature (0 h) and matured (20 h and 44 h) oocytes were used per sample. Proteins were separated on a gradient of pH 3–10 using isoelectric focusing in the first dimension and then by SDS–PAGE in the second dimension. The arrow indicates the CPEB2 form with lowest pI. The experiment was performed three times. The black triangle indicates the molecular weight of CPEB2 protein.

We usedTwo-Dimensional Gel Electrophoresis (2DE) Western blotting to investigate possible post-translational modifications of the CPEB2 protein. As shown in Figure 4, three forms of protein with different pI were present in GV oocytes. We did not observe two distinct signals regarding the molecular weight by this method. After 20 h of in vitro maturation, we detected another signal with a lower pI, possibly representing the phosphorylation of CPEB2 around the time of GVBD (Figure 4, arrow). In metaphase II, all of the CPEB2 forms decreased and only the two most abundant forms were detected. The decrease in metaphase II corresponded to the results of the 1DE Western blot (Figure 3). Using Scansite 3.0 software, three phosphorylation sites of CPEB2 protein were predicted. The four signals could therefore be the different forms of CPEB2 phosphorylation or other post-translational modifications. However, the exact identification of the modifications of CPEB2 will be the subject of further studies.

### 2.3. CPEB2 Protein Moves from Nucleolus to the Cytoplasm

**Figure 5:**
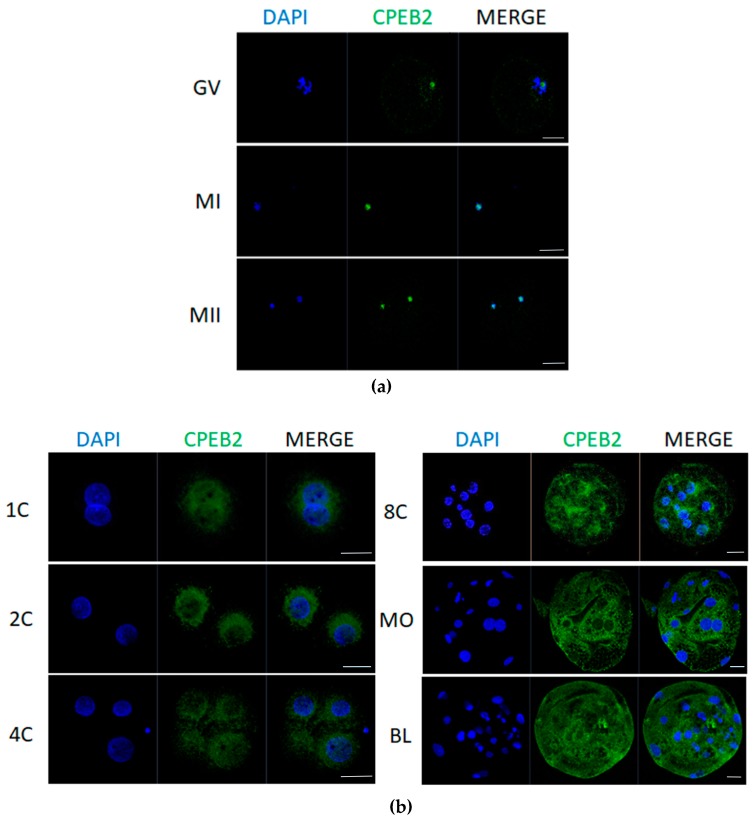
Localization of CPEB2 protein in porcine oocytes and embryos. (**a**) Representative confocal images of porcine GV, MI, and MII oocytes. Scale bars = 50 μm. (**b**) Representative confocal images of porcine pronuclear (1C), two-cell (2C), four-cell (4C), eight-cell (8C), morula (MO), and blastocyst (BL) parthenogenetic embryos. Scale bars = 100 μm. A minimum of 50 oocytes and embryos derived from four independent replicates were analyzed. Micrographs show the ICC detection of CPEB2 (green) and DNA (DAPI, blue).

For the localization of CPEB2 protein we used immunostaining analysis, which revealed that CPEB2 moves from the nucleolus to the cytoplasm. In immature GV oocytes, CPEB2 protein was located in the nucleolus. During maturation in MI and MII stages, CPEB2 co-localized with DNA (Figure 5a) and after parthenogenetic activation it relocated to the cytoplasm in close proximity to the nucleus. This trend persisted in the early developmental stages of embryos until the eight-cell stage, when CPEB2 also started to move into the cytoplasm, away from the nuclei. In the morula and blastocyst stages, CPEB2 expression was significantly stronger and detected throughout the cytoplasm; however, the nuclei were still devoid of CPEB2 accumulation (Figure 5b).

### 2.4. CPEB2 Knockdown Resulted in a Decrease of Properly Maturated Oocytes and Properly Developed Blastocysts

**Figure 6:**
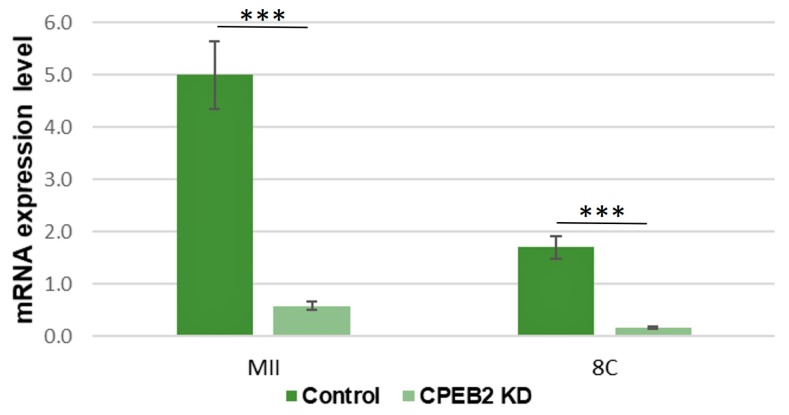
dsRNA efficiency of CPEB2 knockdown (KD) during oocyte maturation and early embryonic development. The relative abundance of CPEB2 mRNA was determined by qRT-PCR in porcine MII oocytes and parthenogenetic eight-cell (8C) embryos. CPEB2 transcript levels were normalized relative to the abundance of GAPDH mRNA (exogenous control) and are shown as means ± SEM (*n* = 4 independent biological experiments with approximately 100 oocytes/embryos in each group). *** indicates statistical difference (*p* < 0.001).

**Figure 7:**
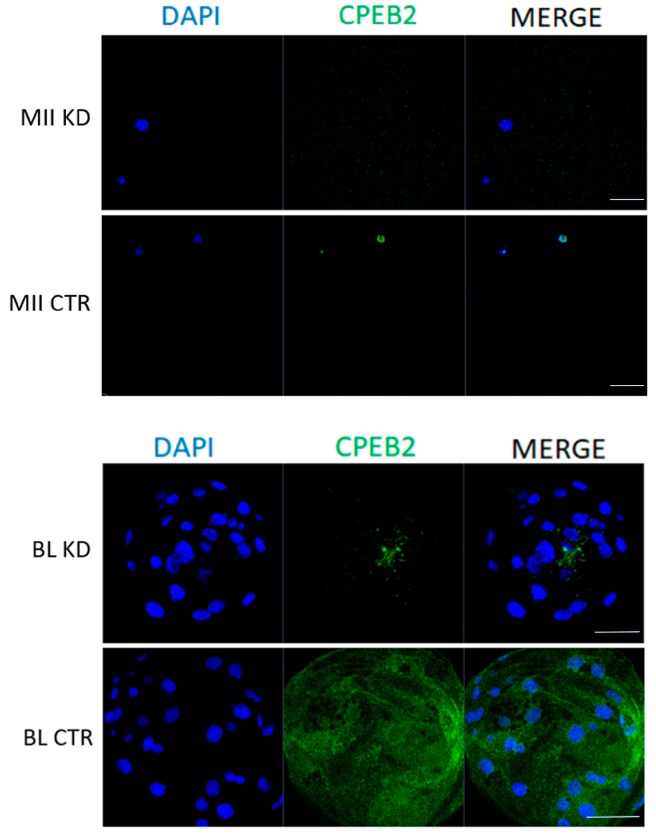
Efficiency of CPEB2 KD during oocyte maturation and embryonic development at the protein level. Representative confocal images show the immunocytochemical detection of CPEB2 (green) protein in porcine MII oocytes and blastocyst (BL). DNA was stained with DAPI (blue). A minimum of 30 oocytes and embryos derived from four independent replicates were analyzed. Scale bars = 100 μm.

To elucidate the function of CPEB2 during meiotic maturation and during embryonic development, GV oocytes and parthenotes were microinjected with CPEB2 dsRNA and incubated in the in vitro cultures. The dsRNA efficiency was measured at the mRNA level by qRT-PCR, which showed 88% and 93% efficiencies of CPEB2 knockdown (KD) in the MII stage and eight-cell stage, respectively (Figure 6). The effectiveness of CPEB2 KD was also confirmed by ICC in the MII and blastocyst stages (Figure 7).

**Figure 8:**
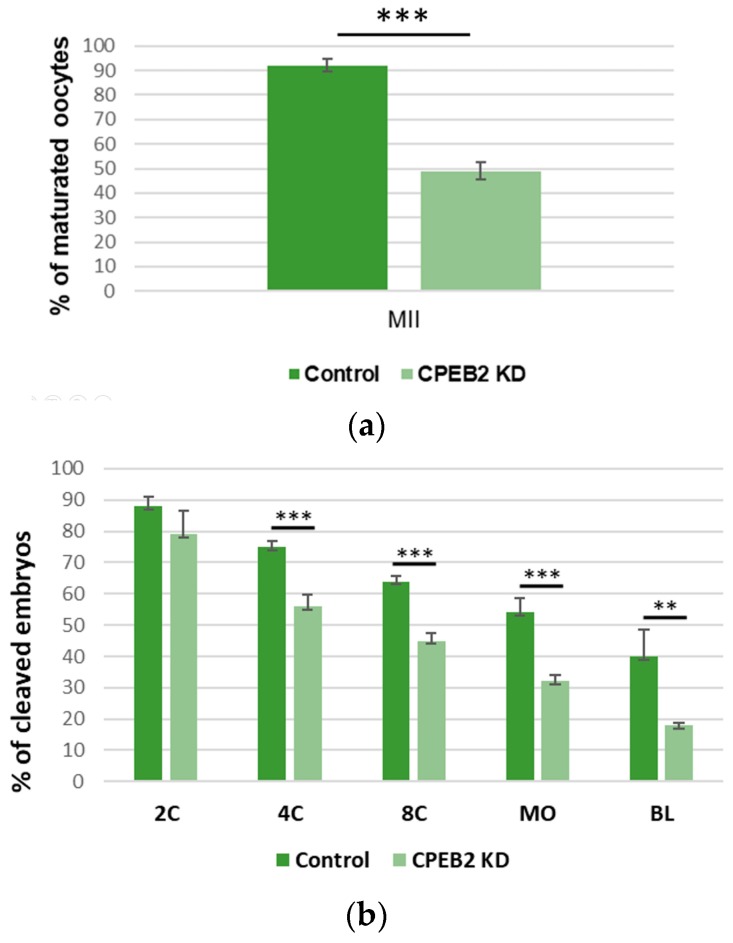
CPEB2 knockdown by dsRNA resulted in a decrease of properly matured oocytes and properly developed blastocysts. (**a**) The effect of CPEB2 KD on porcine meiotic maturation. (**b**) The effect of CPEB2 KD on early porcine embryogenesis including pronuclear (1C), two-cell (2C), four-cell (4C), eight-cell (8C), morula (MO), and blastocyst (BL) parthenotes. Data are means ± SEM (*n* = 4). Asterisks indicate statistical difference (** *p* < 0.01, *** *p* < 0.001).

To evaluate the effect of CPEB2 KD on meiotic maturation and early embryogenesis, MII oocyte and embryo developmental rates were examined. The percentage of MII oocytes in the CPEB2 KD group (43%) was significantly lower (*p* < 0.001) than that in the control group (92%, Figure 8a). At the beginning of embryogenesis there were no significant differences between the control groups and the CPEB2 KD groups. In the two-cell stage, 88% (control) vs. 85% (KD) developed embryos were observed. However, during the four-cell stage the rate was 75% vs. 68% developed embryos, in the eight-cell stage the rate was 65% vs. 45%, and in the morula stage the rate was 54% vs. 28%, with all differences being statistically significant (*p* < 0.001). In the blastocyst stage, we also observed significant differences (*p* < 0.01) between the control group (40%) and the KD group (15%, Figure 8b).

**Figure 9:**
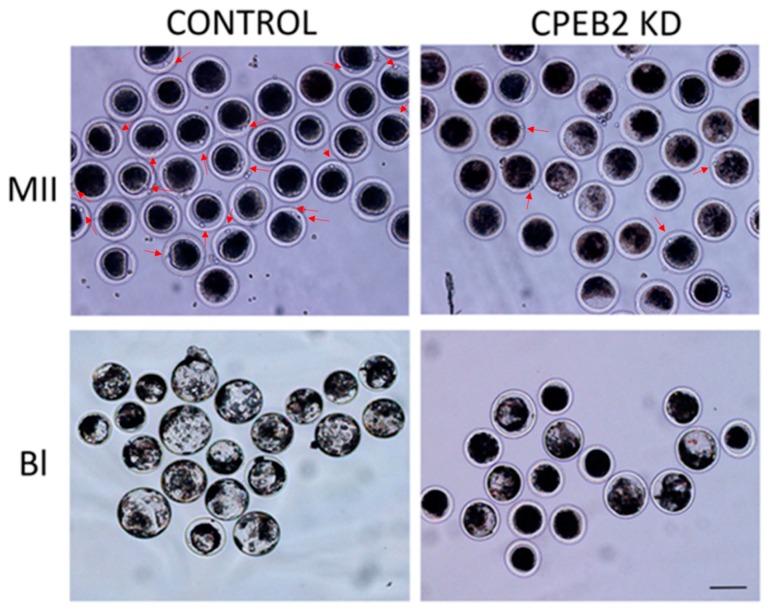
Morphological effect of CPEB2 KD. CPEB2 KD by dsRNA resulted in a decrease of the first polar body extrusion in MII phase porcine oocytes as well as a decrease in porcine parthenogenetic blastocyst formation and size (BL). The red arrows indicate the polar bodies. Scale bar = 200 μm.

Morphological differences between the control and KD groups were also observed. Not only did the CPEB2 knockdown result in an effective decrease of the first polar body extrusion and blastocyst formation, but also the blastocysts from the CPEB2 KD group were significantly smaller than those in the control group (Figure 9).

## 3. Discussion

Cytoplasmic polyadenylation element binding proteins (CPEBs) are a family of proteins that bind to a defined group of mRNAs and regulate their translation. The role of CPEB-mediated translation control has been confirmed in a number of physiological and pathological processes including cell division, germ-cell development, cell differentiation, cellular senescence, synaptic plasticity, learning, and memory [16,31,32,33,34,35,36].

Apart from a brief description of CPEB2 in the nervous system [24,37] and other tissues [23,25,27], not much is known about its expression and role during oocyte meiosis and early embryogenesis. In this study, we confirmed for the first time the CPEB2 mRNA and protein expression, as well as their localization during meiotic maturation in pig oocytes and during early embryogenesis, and their importance in the regulation of these processes.

We detected two splice variants of CPEB2 mRNA in porcine oocytes. All CPEBs are the subjects of alternative splicing, which results in the expression of more splicing variants [24]. Wang and Cooper [15] analyzed mice and human orthogenes of CPEB1–4 proteins in silico and all possessed a large number of transcription variants, leading to the expression of different isoforms of these proteins. They described two RNA splicing variants of CPEB2 in the cells of the mouse retina. The shorter variant (CPEB2A) is formed by the omission of exon 4, and is 90 nucleotides less than the longer variant (CPEB2B). Although two variants of CPEB2 are also present in porcine oocytes (Figure 2), the partial sequencing of the PCR products confirmed only the sequence corresponding to the longer (CPEB2B) variant. The sequencing data did not confirm the presence of the CPEB2A variant; however, the shorter variant may be the result of alternative splicing omitting different exons than in the case of CPEB2A. Wang and Cooper [15] also further mentioned the existence of a long CPEB2 transcript, which encodes a protein almost double in size (109.8 kDa in the human and 107.4 kDa in the mouse ortholog). This transcript has a longer coding sequence at the 5′end, therefore it could not be captured by our proposed primers. The presence of the longer variant in porcine oocytes thus cannot be confirmed or refuted. 

Johnson et al. [28] observed antagonism between these two CPEB2 splice variants and hypothesized their opposing functions—CPEB2B may simply act to bind specific mRNAs and block them from associating with CPEB2A, which inhibits mRNA polyadenylation and translation. They also propose a mechanistic explanation, that the inclusion of the additional 30 amino acids in the full-length CPEB2B changes its function, so that the factor now presents the bound RNA for efficient polyadenylation versus inhibiting the polyadenylation when bound to CPEB2A. Although we documented the presence of two CPEB2 isoforms in porcine oocytes at the mRNA level, we detected only one protein signal of the corresponding size (approximately 50 kDa) using both 1D and 2D Western blotting. The expected difference in size between CPEB2A and CPEB2B proteins is only 2 to 3 kDa. We assume that either the difference is too small to be distinguished under our conditions or only one variant is predominantly expressed in the porcine oocytes. The exact role of the two variants of CPEB2 in porcine oocytes remains to be ascertained.

Wang and Cooper [15] also identified several motifs in the CPEB2 protein, including theoretical phosphorylation sites and the motif that leads to protein interaction with cadherin-1 (CDH1) and cell-division cycle protein 20 (CDC20) components of anaphase-promoting complex/cyclosome (APC/C). This may lead to the degradation of this protein through the ubiquitin-proteasome system, which correlates with our results. The CPEB2 protein was stable until metaphase I, when its partial degradation occurred. This matches with its paralog CPEB1, the degradation of which is necessary for the transition between metaphase I and metaphase II in *Xenopus* oocytes [19], and which has also been observed also in porcine oocytes [38]. Mendez et al. [19] reported similar results, suggesting that the degradation of CPEB1 is necessary for the transition between metaphase I and metaphases II in *Xenopus* oocytes. These results are also supported by us using a more sensitive method of qRT-PCR, demonstrating that the relative expression of CPEB2 increases from the GV to the MI stages and subsequently decreases. Whether the degradation of CPEB2 after metaphase I is essential for the commencement of metaphase II, similar to the case of CPEB1, is a subject for further study.

We confirmed that CPEB2 is also expressed during early porcine embryogenesis. After parthenogenetic activation, CPEB2 expression is still detectable in all developmental stages and, except for a slight increase in the four-cell stage, continuously decreases. Considering that embryonic genome activation occurs at the four-cell stage in porcine embryos [39], these results suggest that porcine CPEB2 is not just a maternally but also a zygotically expressed gene in the later stages of embryo development.

As regards CPEB2 post-translational modification, we detected four specific signals through 2DE Western blot analysis, which could theoretically correspond to phosphorylation at the presumed phosphorylation sites. This phosphorylation may play a role in CPEB2 binding to mRNA and in the regulation of mRNA translation, similar to the role of phosphorylation in CPEB1 [37]. It may also lead to CPEB2 degradation after metaphase I, as reported above. However, the real character and significance of CPEB2 modifications are yet to be elucidated. 

The immunocytochemistry results (Figure 5) show that CPEB2 moves from the nucleolus to the cytoplasm, where it is relocated after parthenogenetic activation. This relocation is probably a response to calcium-mediated signaling and Ca^2+^/calmodulin-dependent protein kinase II (CaMKII) activity. CPEB2–4 have conserved nuclear export signals that are not present in CPEB1 [40].

Not much is known about CPEB2 function and its target mRNA interactions; however, due to the similar levels of protein expression, as well as to the similar primary sequences between CPEB1 and CPEB2, it can be envisaged that CPEB2 shares many similar functions with CPEB1. An overlap in RNA binding specificity between CPEB1 and CPEB2 also indicates the interaction of CPEB2 with β-catenin and (CaMKII) [24], which are both established CPEB1 targets [16,41]. β-catenin is an important mediator of the WNT pathway. Balanced WNT signaling in germ cells is essential for their proliferation [42] and commitment to meiosis, which suggests a requirement for this pathway in follicle/oocyte functions. Overactive β-catenin in female germ cells causes abnormal WNT/β-catenin signaling, which in turn leads to defects in female fertility [43]. In addition, CaMKII is an important regulator of the meiotic cell cycle and spindle assembly, which may exert its effect via the regulation of M-phase promoting factor (MPF) and mitogen-activated protein kinases (MAPK)/ribosomal protein S6 kinase A1 (p90rsk) activity during the meiotic maturation and activation of pig oocytes. After CaMKII blocking, first polar body emission was inhibited [44]. Due to the fact that CPEB2 interacts with these important meiotic regulators, it can be assumed that poor meiotic maturation in CPEB2 KD oocytes might be a response to the disruption of these mechanisms. 

The importance of CPEB-mediated translation regulation during oocyte and sperm development has been clarified by CPEB1 knockout (KO) in a mouse model. These CPEB1 KO mice have no apparent defect in viability but their reproductive organs are reduced (the mice have no detectable ovaries and their testis are 30% smaller in CPEB1 KO) and both males and females are sterile [45]. Moreover, their oocytes have devastating defects during meiotic maturation [46]. Similarly, Stebbins-Boaz et al. [47] showed that *Xenopus* oocytes injected with anti-CPEB1 antibodies fail to polyadenylate and activate c-mos mRNA.

However, unlike in the case of CPEB1 knockout mice, CPEB2 KO mice are fertile, although the number of offspring is significantly reduced. During in vitro maturation, only 42% of the oocytes from these mice are able to reach the MII stage [48], which is consistent with our results for CPEB2 KD porcine oocytes. Nairismägi et al. [49] reported that CPEB2 protein interacts with the TWIST1 transcription factor and negatively regulates its expression. In embryos, TWIST1 mRNA precedes protein expression in epithelial somites and is an essential gene involved in early mesoderm development and dorsal-ventral patterning [50,51]. This could also be the reason for the abovementioned offspring reduction and it can be assumed that only a small percentage of the already low number of well-developed CPEB2 KO/KD porcine blastocysts could further develop.

The disturbed early embryonic development of CPEB2 KD embryos into the blastocyst stage can also be explained by the potential involvement of CPEB2 in tight junction (TJ) assembly. CPEB-mediated TJ has been previously identified in epithelial cell polarity. Normal cells show strong polarity, prevalent cavity formation, and selective permeability in contrast with CPEB1-deficient cells, which have reduced polarity and cavity formation in addition to increased permeability [52,53]. As already documented by Nagaoka et al. [53], CPEB1 plays an important role in regulating the localization of Tjp1 mRNA and thereby in the regulation of cellular tight junctions, which is essential for successful embryonic development. In addition to Tjp1, β-catenin is also connected to TJ regulation [54]. The hypothesis that CPEB2 regulates tight junction biogenesis during embryonic development remains to be proved by further research.

## 4. Materials and Methods

### 4.1. Chemicals

All chemicals used in this research were purchased from Sigma-Aldrich Chemical Company (St. Louis, MO, USA) unless otherwise noted.

### 4.2. Oocyte Collection and In Vitro Maturation 

Porcine ovaries were obtained from gilts at an unknown stage of their estrous cycle from local slaughterhouses (Jatky Český Brod a.s., Český Brod, Czech Republic) and transported to the laboratory within 2 h after slaughter in a physiological saline (0.9% sodium chloride) solution at 36–37 °C.

Ovaries were washed three times in fresh physiological saline and then kept at 38 °C for 15 min. Fully-grown cumulus-oocyte complexes (COCs) were aspirated from follicles (3–6 mm in diameter) by needle (0.80 × 50 mm, Sterican, B. Braun) into a syringe (10 mL, Lauer solo, B. Braun). COCs were transferred into a 14-mL round tube (SPL, BioLab, Singapore) and precipitated for 15 min in a water base at 37 °C. After washing three times in HEPES solution, COCs were selected and only oocytes with granulated cytoplasm and compact cumulus mass were collected for further study. Collected oocytes were washed three times in culture medium M199 (Life Technologies, Carlsbad, CA, USA) supplemented with 10% fetal bovine serum and 0.8 IU/mL serum gonadotropin and chorionic gonadotropin (P.G. 600, Intervet international GmbH, Unterschleißheim, Germany). For maturation, the oocytes were put into a 4-well dish (SPL, BioLab, Singapore) with the same culture medium mixture and covered with mineral oil and incubated for 20 h (GVBD stage), 28 h (MI stage), and 44 h (MII stage) at 38.5 °C in a humidified atmosphere of 5% CO_2_ in air. 

Before sampling for WB and RNA isolation, the oocytes were denuded in HEPES solution supplemented with 1 mg/mL hyaluronidase, then washed three times in HEPES and once in RNase-free H_2_O and stored at −80 °C until use.

### 4.3. Parthenogenetic Activation and Embryo Development 

Denuded MII oocytes with obvious first polar bodies (Pb) and uniform ooplasm were selected for parthenogenetic activation. They were washed three times in HEPES, three times in PBS supplemented with 0.1% BSA (PBS–BSA), and once each in 33% activation mannitol medium (ACT, 280 mM mannitol solution containing 0.001 mM CaCl_2_ and 0.05 mM MgSO_4_) in PBS-BSA, 66% ACT in PBS-BSA, and 100% ACT. After washing, the oocytes were transferred to a fusion chamber containing two electrodes overlaid with ACT. Membrane fusion was induced by applying two DC pulses of 160 V/mm for 60 μs with an Electro Cell Manipulator 2001 (BTX, Inc., San Diego, CA, USA). The embryos were then washed in 66% ACT in PBS-BSA, 33% ACT in PBS-BSA, three times with PBS-BSA solution, and finally in porcine zygote medium-5 (PZM-5). After 3 h of cultivation in PZM-5 supplemented with 7.5 µg/mL of cytochalasin B, the embryos were washed in PMZ-5 medium containing 0.4% BSA and then transferred into fresh medium under mineral oil for 1 day (two-cell stage), 2 days (four-cell stage), 4 days (eight-cell stage), 5 days (morula stage), and 7 days (blastocyst stage) at 38.5 °C in a humidified atmosphere of 5% CO_2_ in air. 

Before sampling, the embryos were washed three times in HEPES and once in RNase-free H_2_O. They were then stored at −80 °C until use.

### 4.4. dsRNA Preparation and Microinjections 

Both CPEB2 RNA isoforms were knocked down by a microinjection of CPEB2 dsRNA. Control groups of oocytes and embryos were microinjected with a corresponding volume of sterile nuclease-free H_2_O. The porcine *CPEB2* dsRNA was amplified in vitro from mRNA of CPEB2. After the construction of primers (*dsCPEB2* forward primer: 5′-TAA TAC GAC TCA CTA TAG GGA GAC CAC GAG CTA TCC ACAC CCA GGAA-3′ and reverse primer: 5′-TAA TAC GAC TCA CTA TAG GGA GAC CAC GGA GAA AGC AAC TCG ACC AG-3′), cDNA was prepared using a Phusion High-Fidelity DNA Polymerase kit (Thermofisher, Deutsch, Germany. In the next step, cDNA was purified using a gel extraction kit (GeneAll Biotechnology, Seoul, Korea). Transcription from PCR products was carried out using a MEGAscript T7 kit (Ambion, Austin, TX, USA) in accordance with the manufacturer’s instructions. Then the dsRNA was purified from the DNA template and single-stranded RNAs by treatment with DNase I and RNase A. Purified dsRNA was eluted in RNase-free water. The concentration was determined by measuring the optical density at 260 nm (Nanodrop, Thermofisher, Deutsch, Germany) and adjusted to a final concentration of 1 µg/µL dsRNA aliquots was stored at −80 °C. 

Subsequently, 5–10 pl of dsRNA was microinjected into the cytoplasm of denuded oocytes in the GV stage, or into parthenogenetic zygotes 8 h post activation. For the injection, Eppendorf FemtoJet microinjector (Eppendorf, Hamburg, Germany) was used together with a Nikon TE2000-U inverted microscope (Nikon Corporation; Tokyo, Japan). 

After microinjection, the oocytes were cultured for 24 h in the standard cultivation medium supplemented with 1 mM dbcAMP. Then the oocytes were washed and cultured further in fresh medium under the same conditions as above. Embryo cultivation was again carried out as already stated. Knockdown efficiency was confirmed by qRT-PCR. All microinjection experiments were performed in quadruplicate, with approximately 100 oocytes/zygotes injected in each group.

### 4.5. Evaluation of Oocyte Maturation 

For the evaluation of maturation stages, two methods were used. Oocytes intended for RNA isolation, immunocytochemistry, and MII maturation rates were washed three times in PBS–BSA, stained for 5 min in 5 µg/µL DAPI (D9542) in PBS–BSA, and then washed in PBS–BSA. The maturation stage of each oocyte was checked under UV light. 

For samples intended for Western blot and 2DE Western blot, a portion of the oocytes was denuded and fixed in a mixture of ethanol and acetic acid solution (3:1) for 48 h. Staining was performed with 1% orcein in 50% aqueous acetic acid and 1% sodium citrate, after which the oocytes were washed with 40% acetic acid. Oocytes were observed using phase-contrast microscopy (Carl Zeiss, Jena, Germany) and only samples from cultivations of more than 85% oocytes at the desired stage were used for further experiments.

### 4.6. Western Blotting 

Samples of 100 oocytes were lysed in 10 µL of 1× Reducing SDS Loading Buffer (Cell Signaling Technology, Danvers, MA, USA) by heating at 100 °C for 5 min. For the separation of proteins, SDS-PAGE was used. Proteins were then transferred to an Immobilon P membrane (Millipore, Bedford, MA, USA) using a semidry blotting system (Biometra GmbH, Goettingen, Germany) for 25 min at 5 mA/cm^2^. Membranes were blocked in 5% skimmed milk dissolved in 0.05% Tween-Tris-buffer saline (TTBS), pH 7.4 for 1 h. After a brief washing in TTBS, membranes were incubated at 4 °C overnight with the following primary antibodies: CPEB2 (ARP41186; Aviva System Biology, San Diego, CA, USA), monoclonal anti-β-tubulin antibody (T4026; Sigma-Aldrich). After washing the membranes 3 × 10 min in TTBS, incubation with a horseradish peroxidase-conjugated donkey anti-rabbit or anti-mouse IgG antibody (Jackson Immuno Research, Suffolk, UK) followed for 1 h at room temperature. An chemiluminescent reagent ECL-plus detection system (GE Healthcare, Chalfont St Giles, Bucks, UK) was used for visualization according to the manufacturer’s instructions. A GS-800 Calibrated Densitometer (Bio-Rad, Hercules, CA, USA) was used to scan the films and Quantity One 1-D Analysis Software (Bio-Rad) was used for quantification.

### 4.7. Two-Dimensional Gel Electrophoresis (2DE)

Samples containing 200 oocytes were lysed in 30 µL of lysis buffer containing 7 M urea, 2 M thiourea, 3% *w*/*v* CHAPS, 2% *v*/*v* Nonidet-P40, and 5 mM TCEP in the presence of inhibitors of proteases and phosphatases (Roche, Basel, Switzerland) and 0.2% ampholyte (Bio-Rad, Hercules, CA, USA) according to the manufacturers’ directions for 15 min at room temperature. Next, 120 µL of rehydration buffer containing 7 M urea, 2 M thiourea, 4% CHAPS, 200 mM DeStreak, inhibitors of proteases, phosphatases (Roche, Basel, Switzerland), 0.2% ampholyte (Bio-Rad, Hercules, CA, USA), and 0.5% bromophenol blue was added and the samples were lysed for 10 min. Isoelectric focusing separation (IEF) was performed on an IEF Cell (Bio-Rad, Hercules, CA, USA) system using the following program: 1 h to 200 V, 1 h to 500 V, 30 min to 1000 V, 30 min to 4000 V, and 4000 V until a total of 8 kVh was reached. After IEF separation, the gel strips were equilibrated in 50 mM Tris, pH 8.8, 6 M urea, 30% glycerol, 4% SDS, 100 mM DeStreak, and a trace of bromophenol blue for 25 min. The 2DE was followed by SDS-PAGE and WB.

### 4.8. PCR 

Total RNA was isolated from groups of 280 oocytes using an RNeasy Micro Kit (74004; Qiagen, Hilden, Germany. For the reverse transcription, SuperScript III Reverse Transcriptase (Life Technologies) was used. PCR was performed with following primers: CPEB2 forward primer—5′- TCA CTA GTT CCT GGG GAG CAA TGC AT-3′ and reverse primer—5′-GCA CTA GTG TTC CAG CGG AAG TGG AT-3′. cDNA was subjected to electrophoresis on 2% agarose gel and stained with GelRed (Biotium, Hayward, CA, USA) or ethidium bromide. Gels were observed and photographed using a Kodak Gel Logic 100/200 Camera (Carestream Health, Inc., Rochester, NY, USA), K. G. L. integrated illuminator cabinet (Carestream Health, Inc.), and KODAK MI SE software (v. 4.5.0.; Carestream Health, Inc.).

### 4.9. qRT-PCR 

For the evaluation of gene expression, mRNA was isolated from 50 oocytes and 25 parthenogenetic embryos (two-cell, four-cell, and eight-cell stages, morula and blastocyst) per group, using a Dynabeads mRNA Direct Kit (61011, Ambion, Life Technologies, CA, USA), and cDNA was synthesized with the HyperScript™ First-Strand Synthesis Kit (601-005, GeneAll Biotechnology, Seoul, Korea) according to the manufacturer’s instructions. For subsequent experiments, a minimum of three replicates of isolated mRNA from each stage were used.

WizPure™ qPCR Master (Super Green) (W1731, Wizbiosolutions, Seongnam, Korea) was used to provide the real-time quantification of target gene CPEB2 and of GAPDH, as a housekeeping gene. CPEB2 and GAPDH were amplified with specific primer pairs (CPEB2 forward primer: 5′-GTT CAG ATC CGT CCT TGG AA-3′ and reverse primer: 5′-GGA GAA AGC AAC TCG ACC AG-3′; GAPDH forward primer: 5′-GGG CAT GAA CCA TGA GAA GT-3’ and reverse primer: 5′-AAG CAG GGA TGA TGT TCT GG-3′) which were designed with Primer Premier 5 (PREMIER Biosoft, CA, USA). qPCR was conducted using the CFX96 Touch Real-time PCR Detection System (Bio-Rad, CA, USA) under the following conditions: denaturation at 95 °C for 10 min; 40 cycles of amplification and quantification at 94 °C for 10 s, 60 °C for 30 s, and 72 °C for 30 s with a single fluorescence measurement; melting at 65–95 °C with a heating rate of 0.2 °C/s and continuous fluorescence measurement. Relative gene expression was quantified by normalization to GAPDH levels using the 2^−ΔΔ*C*t^ method [55]. Four independent experiments were performed with triplicate samples for each stage.

### 4.10. Immunocytochemistry (ICC)

Before staining, the oocytes and embryos were treated for 5–10 s with acidic Tyrode’s solution (T1788) to remove zona pellucida. They were then fixed for 30 min in 3.7% paraformaldehyde. Next, they were washed three times in PVA-PBS and permeabilized with PVA-PBS containing 0.5% Triton X-100 for 30 min. For the following washes, washing solution (0.1% Triton X-100 + 0.01% Tween 20 in PBS) was used three times for 5 min. The oocytes and embryos were blocked in 1% BSA in PBS for 1 h and then incubated overnight at 4 °C in the same solution with the addition of the anti-CPEB2 antibody (dilution 1:50, sc-55622, Santa Cruz, Dallas, TX, USA). In the next step, the oocytes were washed with washing solution for 5 min and then treated with goat anti-rabbit secondary antibody (1% BSA in PBS containing Alexa-Fluor-488, dilution 1:200) for 1.5 h. After washing in washing solution, oocytes and embryos were incubated with 10 mg/mL DAPI in PVA-PBS for 10 min in the dark, washed three times in PVA-PBS, and mounted onto glass slides with mounting media Vectashield (94010, Vector Laboratories, Burlingame, CA, USA) and examined using a confocal microscope (Zeiss LSM 710 META, Jena, Germany). Images were processed using Zen software (version 8.0, Zeiss, Jena, Germany).

### 4.11. Statistical Analysis 

Data were analyzed using SPSS software version 11.0 (SPSS Inc., Chicago, IL, USA). Differences in normalized data across meiotic maturation and developmental stages were determined by one-way ANOVA. Individual mean comparisons were performed using Fisher’s least significant difference (LSD) method. Differences between CPEB2 control and CPEB2 KD groups were statistically analyzed by an independent two-sample Student´s *t*-test. Data are presented as averages ± SEM. The values indicated by asterisks are significantly different (* *p* < 0.05, ** *p* < 0.01, and *** *p* < 0.001).

## 5. Conclusions

For the first time, we have described the expression of CPEB2 at both mRNA and protein levels, as well as its localization during the meiotic maturation of porcine oocytes and during early embryogenesis. Our results indicate that there are two RNA isoforms of CPEB2 in porcine oocytes and that CPEB2 is likely to be post-translationally modified, possibly by phosphorylation. The level of CPEB2 mRNA expression changes during meiotic maturation and early embryogenesis and likely plays an important role for their successful completion, since CPEB2 knockdown by double-stranded RNA reduces the rate of the meiotic maturation of oocytes as well as the rate of development to the blastocyst stage and the blastocyst size.

Further studies will be required to determine how CPEB2 is involved in the signaling pathways regulating meiotic maturation and early embryonic development in addition to which genes it directly affects.

## Supplementary Materials

Supplementary materials can be found at http://www.mdpi.com/1422-0067/19/10/3138/s1.

## Abbreviations

1C	one-cell embryo
1DE WB	one-dimensional gel electrophoresis
2C	two-cell embryo
2DE	two-dimensional gel electrophoresis
2^−ΔΔ*C*t^	double delta threshold cycle analysis
3′-UTR	the three prime untranslated region
4C	four-cell embryo
8C	eight-cell embryo
ACT	activation mannitol medium
CaMKII	Ca^2+^/calmodulin-dependent protein kinase II
ANOVA	analysis of variance
BL	blastocyst
bp	base pair
BSA	bovine serium albumin
CDC20	cell-division cycle protein 20
CDH1	cadherin-1
cDNA	complementary deoxyribonucleic acid
CHAPS	3-[(3-Cholamidopropyl)dimethylammonio]-1-propanesulfonate
c-mos	protein of mos proto-oncogene
COCs	cumulus-oocyte complexes
CPE	cytoplasmic polyadenylation element
CPEB1	cytoplasmic polyadenylation element-binding protein 1
CPEB2	cytoplasmic polyadenylation element-binding protein 2
CPEB3	cytoplasmic polyadenylation element-binding protein 3
CPEB4	cytoplasmic polyadenylation element-binding protein 4
CPEBs	cytoplasmic polyadenylation element binding proteins
CSF	cytostatic factor
CTR	control
DAPI	2-(4-amidinofenyl)-1H-indol-6-carb
dbcAMP	N6,2′-O-Dibutyryladenosine 3′,5′-cyclic monophosphate sodium salt
DNA	deoxyribonucleic acid
dsRNA	double-stranded ribonucleic acid
ECL	chemiluminescent reagent
eEF2	eukaryotic elongation factor 2
GAPDH	glyceraldehyde-3-phosphate dehydrogenase
GV	germinal vesicle
GVBD	germinal vesicle breakdown
HIF-1α	hypoxia-inducible factor-1α
ICC	immunocytochemistry
IEF	isoelectric focusing separation
IgG	immunoglobulin G
KD	knockdown
KO	knockout
LSD	least significant difference
M	marker
MAPK	mitogen-activated protein kinases
MI	metaphase I
MII	metaphase II
MO	morula
MPF	maturation promoting factor
mRNA	messenger ribonucleic acid
NC	negative control
NCBI	National Center for Biotechnology
ORB1	oo18 RNA-binding protein 1
ORB2	oo18 RNA-binding protein 2
ORF	open reading frame
p90rsk	ribosomal protein S6 kinase A1
PAGE	polyacrylamide gel electrophoresis
Pb	polar body
PBS	phosphate-buffered saline
PCR	polymerase chain reaction
pI	isoelectric point
Poly(A)	polyadenylic acid
Poly(C)	polycytidylic acid
Poly(U)	polyuracilic acid
PVA	poly(vinyl) alcohol
PZM-5	porcine zygote medium-5
qRT-PCR	real-time quantitative reverse transcription polymerase chain reaction
RNA	ribonucleic acid
RRM	RNA-recognition motif
RT-PCR	everse transcription polymerase chain reaction
SCP2	synaptonemal complex protein 2
SCP3	synaptonemal complex protein 3
SDS	sodium dodecyl sulfate
SEM	standard error of the mean
SCP1	synaptonemal complex protein 1
SPSS	Statistical Package for the Social Sciences
TCEP	tris(2-carboxyethyl)phosphine
TJ	tight junction
Tjp1	tight junction protein 1
TTBS	Tween-Tris-buffered saline
TWIST1	twist-related protein 1
WB	Western blot
WNT	WNT gene family
SEM	standard error of the mean
SCP1	synaptonemal complex protein 1
SPSS	Statistical Package for the Social Sciences
TCEP	tris(2-carboxyethyl)phosphine
TJ	tight junction
Tjp1	tight junction protein 1
TTBS	Tween-Tris-buffered saline
TWIST1	twist-related protein 1
WB	Western blot
WNT	WNT gene family
