# Supplementary figures and images for: CPEB2 Is Necessary for Proper Porcine Meiotic Maturation and Embryonic Development
Source: Int J Mol Sci. 2018 Oct 12;19(10):3138. doi: 10.3390/ijms19103138 (PMC6214008; doi:10.3390/ijms19103138)

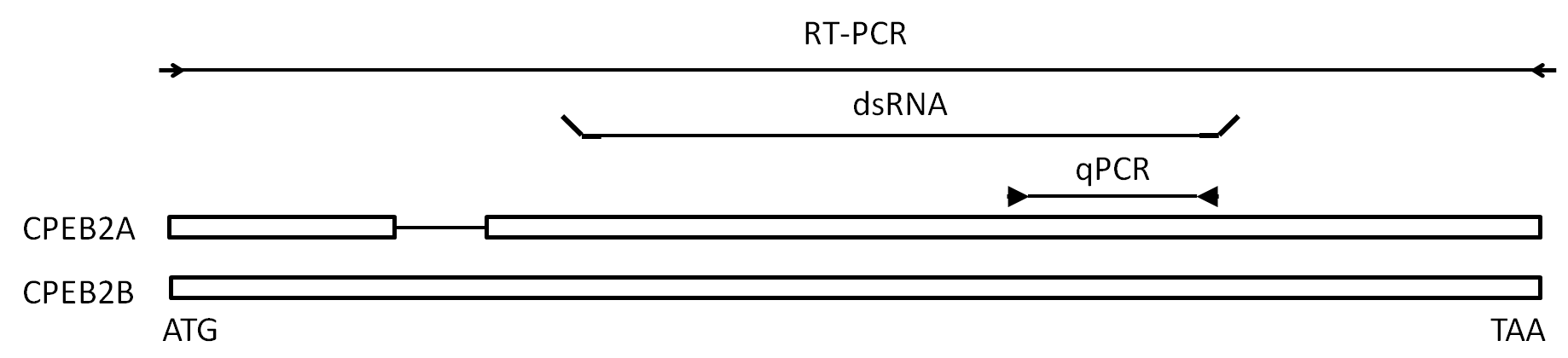

Supplement: Supplementary file 1 [file ijms-19-03138-s001.zip › supplement 1.tif]

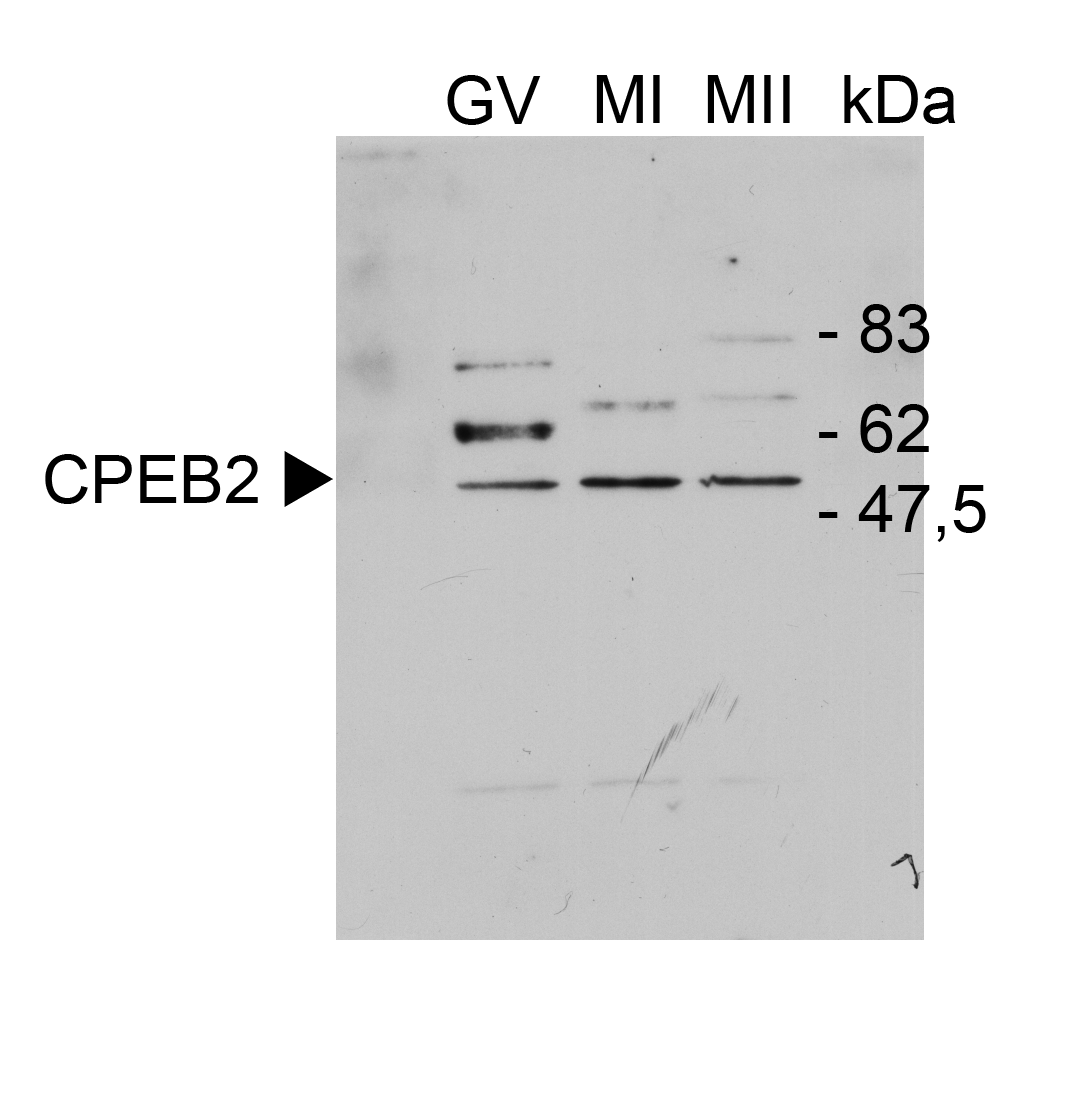

Supplement: Supplementary file 1 [file ijms-19-03138-s001.zip › supplement 2.tif]
